# Differentiating bacteria by their unique surface interactions

**DOI:** 10.1371/journal.pone.0327489

**Published:** 2025-06-30

**Authors:** Nicholas K. Kotoulas, Stephanie Tan, Justin R. Nodwell, M. Cynthia Goh

**Affiliations:** 1 Department of Chemistry, University of Toronto, Toronto, Ontario, Canada; 2 Department of Biochemistry, University of Toronto, Toronto, Ontario, Canada; University of Pennsylvania, UNITED STATES OF AMERICA

## Abstract

New, rapid, and accessible approaches to bacterial detection are necessary to help curb the rising impacts of antimicrobial resistance. In this study, we introduce a technique that distinguishes bacteria through their unique surface interactions. By measuring and combining the interaction strengths of a bacterium across a set of chemically defined surfaces, we produced a novel bacterial identifier termed the surface interaction profile (SIP). The interaction strengths of twelve test bacteria across three discrete polyelectrolyte multilayer films (PEMs) were measured, facilitated by introducing each bacterial suspension to individual PEMs in microfluidic channels over a 10-minute interaction period and rinsing to remove bulk and loosely bound bacteria. The remaining surface-bound cells were counted via microscopy and plotted against suspension concentrations to build attachment curves whose slopes were measured as the strength of interaction for a given bacteria-PEM combination. These slopes were collected, per bacterial type, to produce each SIP. SIPs were capable of distinguishing between our pathogenic strains (*Klebsiella pneumoniae, Acinetobacter baumannii, Pseudomonas aeruginosa, Enterococcus faecalis*, methicillin-resistant *Staphylococcus aureus*, and vancomycin-intermediate *Staphylococcus aureus*) by Gram stain and individual species, and each blind test pathogen was successfully identified through SIP comparison. Furthermore, SIPs were also successful at differentiating between select *Staphylococcus aureus walKR* mutants impacting cell wall metabolism and high-risk antibiotic resistance mutants (MRSA and VISA), highlighting the utility and future diagnostic potential of this technique.

## Introduction

Rapid bacterial diagnostics are essential in the global response to rising antimicrobial resistance (AMR). In principle, improving the turnaround time for bacterial identification and antibiotic susceptibility testing (AST) enables doctors to quickly prescribe effective antibiotics, improving patient outcomes while reducing the overuse and misuse of antibiotics that contribute to the further breeding of resistance [1,2]. In practice, traditional approaches (e.g., culture-based, phenotypic, turbidimetric) and improved or emerging rapid techniques (e.g., nucleic acid amplification tests, mass spectrometry, enhanced turbidity) are either significantly time-consuming or resource-intensive [3]. Furthermore, emerging technologies are limited in their routine use and implementation in low-resource settings due to significant cost and operational barriers [4,5]. A chorus of risk factors, including limited access to clean water and sanitation, poor infection control practices in healthcare facilities, and a lack of antibiotic stewardship, increase the prevalence of antibiotic-resistant infections, associated mortality rates and healthcare costs in developing countries [6,7]. Continued progress toward new, rapid, and accessible approaches to pathogen detection is necessary to curb the global impact of AMR by improving diagnostics and infection surveillance practices beyond our borders.

This study introduces a novel technique that differentiates bacteria based on their surface interactions, with the aim of developing a more rapid and less resource-intensive bacterial diagnostic. Variability in bacterial surface composition is observed across classes of bacteria, from broader differences between Gram stain and species to more subtle differences between strains and individual mutants, which impact the expression of virulence factors and/or antibiotic resistance [8–13]. Their unique surface composition and chemistry directly influence how they interact with surfaces [14,15], including those in their natural environments. The early stages of attachment are facilitated predominantly by electrostatics and, to a lesser extent, van der Waals forces and hydrophobic interactions [16–18]. In this context, the bacterial surface becomes a viable, versatile target for detection, offering a broad spectrum of potential surface interactions that can be utilized to differentiate between a wide array of bacteria.

**Fig 1:**
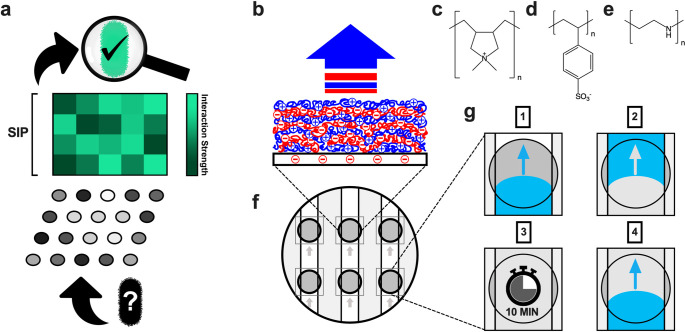
Surface interaction profiles (SIPs) and experimental design. a, A schematic depicting the SIP, a novel bacterial identifier produced by combining interaction strengths between a given bacterium and a series of chemically unique surfaces. b, An illustration of a polyelectrolyte multilayer film assembled via the layer-by-layer approach. c-e, The chemical structures of polydiallyldimethylammonium chloride (PDDA), polystyrene sulfonate (PSS), and polyethylenamine (PEI). f, A schematic of six test PEM surfaces (grey circles) encased in microfluidic channels (squares) in a polystyrene Petri dish. g, The protocol for bacterial-surface interactions: (1) pre-soaking PEM with PBS buffer (30 min), (2) introducing bacterial suspensions to each channel, (3) leaving them in contact for 10 minutes, and (4) rinsing channels with PBS.

The attachment of bacterium A and B to an identical substrate C may not yield a measurable difference in their interaction strengths to differentiate them, primarily due to the small pool of possible interactions available when using a single substrate as a probe. Increasing the number and chemical diversity of substrates for which interaction strengths are measured enhances the capacity and complexity of potential interactions used to differentiate. In principle, this would allow for more nuanced variations in surface composition to be detectable, improving the probability of distinguishing bacterium A from B while also expanding the potential utility beyond species- or strain-level identification. By integrating interaction strengths across multiple chemically distinct surfaces we generate a unique bacterial identifier, the surface interaction profile (SIP) (Fig 1), designed to differentiate bacteria by direct comparison and to identify a clinical pathogen causing infection through comparison across a library of pre-measured SIPs.

To test the distinguishing power of SIPs, we quantified the surface interaction strengths of twelve test bacteria across three discrete, chemically distinct polyelectrolyte multilayer (PEM) films (Fig 1b) topped with the following positive- and negative-charged polymers: poly(diallyldimethylammonium chloride) (PDDA) (Fig 1c), polystyrene sulfonate (PSS) (Fig 1d), and polyethylenimine (PEI) (Fig 1e). All PEMs were encased in individual microfluidic channels (Fig 1f) and were exposed to four serially diluted bacterial suspensions (prepared via three biological replicates) through the following steps (Fig 1g): (1) pre-soaking the PEMs, (2) introducing bacterial suspensions to the channels, (3) leaving them in contact for 10 minutes, and (4) removing excess and loosely bound cells via rinsing. The remaining surface-attached bacteria were counted via optical microscopy and plotted against all suspension concentrations to build attachment curves (S1 Fig). Their slopes represented the interaction strength values for each bacterial-PEM combination and were collected (per bacterium) to produce each SIP (Fig 1g). A set of pathogens responsible for the majority of nosocomial infections [19] was tested to compare SIPs of bacteria broadly by Gram stain and individual species, including a blind experiment. This was followed by comparing SIPs across *Staphylococcus aureus* mutants affecting cell wall metabolism and antibiotic resistance (including single- and multi-drug-resistant mutants).

## Methods

### Polymer stock solutions

The stock solutions (10 mg/mL) for polydiallyldimethylammonium chloride (PDDA) (Aldrich, 208.00 mg/mL, M_w_ 100,000–200,000), polyethylenimine (PEI) (Aldrich, 50% wt, M_w_ 750,000), and polystyrene sulfonate (PSS) (Aldrich, 344.13 mg/mL, M_w_ 200,000) polyelectrolytes were prepared by weight and dissolved in deionized (DI) water (pH 6.998, Millipore Milli-Q) with magnetic stirring. Three working polymer solutions (1 mg/mL) for each polymer type were prepared by diluting each respective stock (x10) with DI water and were sonicated (10 minutes) prior to use.

### Layer-by-layer assembly of PEMs

Using a template (S3 Fig a), thirty-six small circles (1 cm diameter) were drawn across the interior of six Petri dish lids that formed the perimeter of each individual PEM surface. Three polyelectrolyte multilayer (PEM) type surfaces were prepared via the layer-by-layer approach with the following combinations of positive and negative polymer layers: PDDA/PSS (5.5 bilayer, positively charged PDDA-topped PEM), PDDA/PSS (5 bilayer, negatively charged PSS-topped PEM), and PEI/PSS (5.5 bilayer, positively charged PEI-topped PEM).

To begin, a small aliquot (100 µL) of the positively charged polymer was added to each marked circle: PDDA (1 mg/mL) for PDDA- (N = 12) and PSS-topped (N = 12) PEMs, and PEI (1 mg/mL) for the PEI-topped PEM surfaces (N = 12). Once the positive polymer solution was added to each circle (N = 36), they were left to interact for 1 minute. After the interaction period, each individual surface was rinsed from top to bottom (unidirectional flow) in the order that the polymer was added (~2 seconds duration per surface) with a light stream of DI water: the rinsing protocol is detailed in S4 Fig. With a single Petri lid held at an approximately 35˚ angle (a), three of six surfaces were rinsed individually from left to right (b) with the DI water waste collecting at the bottom lip of the lid. The lid was then rotated counterclockwise by 180˚ (c), maintaining its angled position and allowing the excess water to flow around the lid edge. The final three surfaces were rinsed from above in the same order (left to right) as above (d), and the accumulated DI water was subsequently poured out (e). The lids were then flipped upside down and firmly tapped eight times against three piled Kimwipes on the lab bench, removing excess DI water without contacting the PEMs (f). This process was repeated for all remaining surfaces.

A small aliquot (100 µL) of PSS (1 mg/mL) was then added to all 36 surfaces in sequence and left to incubate for 1 minute. The same rinsing protocol (above) was used post-incubation. This cycle of polymer addition and rinsing was repeated a total of 9 times for the (+) PEMs and 8 times for the (-) PEMs. The polymer incubation period for the last two layers of each PEM type (layers 10 and 11 of the (+) PEMs and layers 9 and 10 for the (-) PEMs) were left to incubate for 5 minutes. After the final rinsing step, when all polyelectrolyte layers had been added, the surfaces were left to dry with the lids ajar.

### Building the fluidic chip

Four strips of double-sided tape (1/2 in. double-sided polyester film tape, Tesa) were cut (6 cm long), and one strip (i) was added between two of three PEM surfaces in each row (S3 Fig b). On either side of the first strip of tape, two more strips (ii and iii) were added parallel to the first at 0.7 cm on either side. Finally, the last strip (iv) was added with the same 0.7 cm spacing to the left of strip iii. This was repeated for all six Petri dish lids.

Next, the top plastic layer of each strip of tape was carefully removed using a tweezer. Each PEM surface was topped with a thin glass coverslip (1.5x1.5 cm, VWR), where the PEM was positioned at the center, and the coverslip edges contacted the double-sided tape strips on either side (S3 Fig b). Pressure was applied by hand at all points of contact between the cover slip and tape. All chip inlets and the inlet-facing edge of all coverslips had hydrophobic boundaries drawn on via a black permanent marker (S3 c).

### Preparing cell suspensions

Bacteria were first prepared by inoculating the strain of interest and incubating it overnight with shaking (220 RPM) at 37˚C in LB. Bacteria were sub-cultured the next day at 1:1000 into fresh LB and incubated with shaking at 37˚C for 4–6 hours to the desired optical densities measured at 600 nm (OD_600_). The specific species, strains, mutants, and OD_600_ measurements pre- and post-resuspension in phosphate-buffered saline (PBS, pH 7.4) can be found in S1 Table. All test bacteria were laboratory stock, and all *Staphylococcus aureus walKR* mutants were sourced from a previous study [20]. *S. aureus* ATCC29213 *walR* EV and *walR murOP* were both grown in LB containing 12 µg/mL chloramphenicol, and methicillin-resistant *Staphylococcus aureus* (MRSA) CA-USA300 ∆*tarO*-10 was grown in LB containing 300 µg/mL spectinomycin.

Next, two 1 mL aliquots were transferred to individual Eppendorf tubes and centrifuged at 8000 x g for 3 minutes to obtain a pellet for each strain. The supernatant was removed, and the pellet was resuspended with 500 µL PBS. The 500 µL suspensions of two tubes from the same original culture were combined to produce a concentrated suspension (1 mL) each. The cells were rinsed two more times with PBS. Subsequently, all concentrated PBS suspensions were further serially diluted two-fold to produce four unique dilutions per suspension labelled C1-C4 from lowest to highest cell concentration. All bacteria were tested using three biological replicates, where each suspension was prepared from a single colony.

### Bacterial-PEM interactions

All fluidic chips were arranged by PEM type in a biosafety cabinet with Petri covers removed. Groups of three PEMs in a row were marked C1-C4, covering all cell suspension concentrations per PEM type (N = 3 replicates per concentration per PEM type). The steps involved in the addition of a bacterial suspension to a fluid lane are illustrated in S5 Fig. Once the same-day bacterial suspensions reached the desired range of optical densities, all 36 fluid lanes were pre-wet with 100 µL of PBS for 30 minutes (1). Post-wetting, excess PBS buffer was removed via Kimwipe contact at both the fluid lane inlets and outlets while preventing the removal of PBS from the fluid lane interiors. Aliquots (70 µL) of C1-C4 cell suspensions were pipetted onto the marked lane inlets (N = 36), one suspension per lane per PEM type in triplicates, and were wicked through in sequence via Kimwipes in contact with the lane outlet (2). The suspensions were left to interact for 10 minutes (3). Post-interaction, all lanes were rinsed with a 100 µL aliquot of PBS buffer in the sequence that the suspensions were wicked through initially (4). This step was repeated for a total of 200 µL PBS wicked through using a fresh Kimwipe per lane.

### Surface-bound cell imaging and processing

With all lanes rinsed, each PEM surface (N = 36) was imaged 10 times per surface (N = 360 total images per type of bacterium) using an inverted microscope (S5 Fig). All images were taken at 200x magnification, and the positions along each surface were selected randomly (S6 Fig). Areas containing large pieces of dust or debris were avoided.

All images were batch processed in ImageJ (open source) to count the surface-bound cells: the left and right edges were cropped out, followed by applying the Find Maxima function (15 prominence, excluding edge cases) to extract cell counts. The batch processing macros code is as follows:

makeRectangle(503, 1, 925, 1079);

run(“Crop”);

run(“Find Maxima...”, “prominence=15 exclude output=Count”);

### Building attachment curves and surface interaction profiles (SIPs)

The mean cell counts across replicates per added bacterial suspension concentration (OD_600_) were plotted to produce attachment curves (mean ± SEM) per PEM type per bacterium (S2 Fig). All cell counts across all replicates were plotted per added bacterial suspension concentration (OD_600_) and a linear regression was applied. The interaction strength values (slope values) per bacterial-PEM combination were collected per bacterial type to produce each bacteria-specific SIP.

## Results and discussion

Polyelectrolyte multilayer (PEM) films were selected as the probe substrates in this study due to their simple and highly reproducible fabrication process, the layer-by-layer assembly (Fig 1b), which yields robust thin films with easily adjustable surface properties [21]. The PEM surface properties (e.g. charge, hydrophobicity, roughness) are determined by the deposition conditions (e.g. pH, ionic strength) and the polymer type, primarily in the topmost exposed layer [22,23]. Each individual PEM offers a variety of interactions from the charged (positive or negative), uncharged, hydrophobic and hydrophilic moieties present in the polymers used, expanding the pool of interactions needed while maintaining a small number of probing substrates. The individual polymer layers are deposited under equilibrium conditions, such that while they are structurally amorphous, the distribution of these distinct chemical features is reproducibly uniform. All fabricated PEMs used in this study were pre-wet with phosphate buffer saline (PBS) for 30 minutes to allow them time to reconfigure through polymer relaxation and to prevent this process from impacting the bacterial-PEM interactions. Bacteria were introduced as liquid suspensions in PBS to ensure that they remained viable throughout the duration of each experiment. The suspensions were introduced to each PEM through individual microfluidic channels (Fig 1f) that were designed to limit variability in fluid flow rate and direction. Shear stress caused by fluid flow can significantly impact bacterial attachment [24], requiring consistent control over the hydrodynamics of our channels.

**Fig 2:**
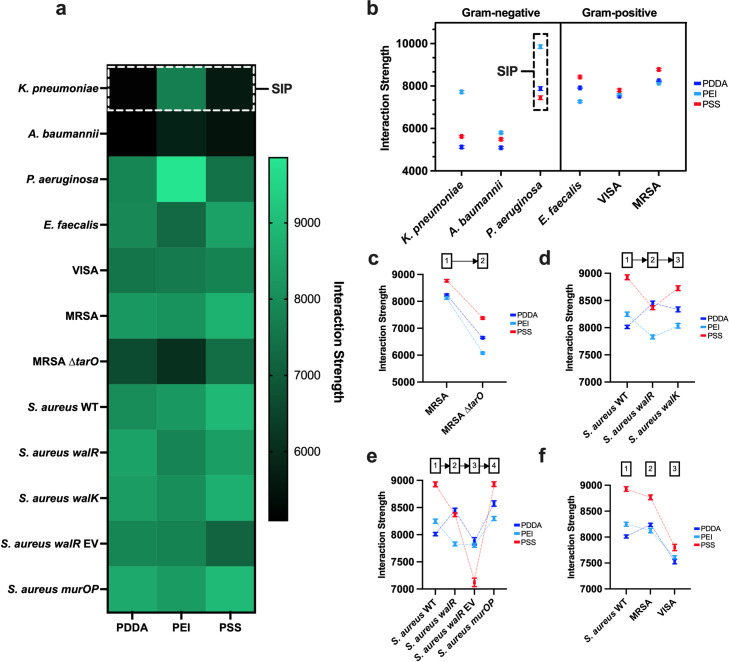
Surface interaction profiles (SIPs) of all twelve test bacteria. a, The interaction strengths of all twelve test bacteria plotted as a heatmap for SIP comparison. b, The SIPs organized by Gram stain (mean ± SEM). c-f, All SIPs across *S. aureus* mutants (mean ± SEM).

The SIPs of all test bacteria were plotted as a heat map (Fig 2a), highlighting distinct differences in the relative magnitudes of their interaction strengths across each PEM. While grouping the pathogen SIPs by Gram stain (Fig 2b), we observed the Gram-negative bacteria as having a stronger attachment preference for PEI while the Gram-positive bacteria preferred PSS. This was unsurprising, given that these two classes represent broad differences in bacterial envelope structure and surface chemistry, including polymer layers that encompass the cell. They comprise lipopolysaccharides (LPS) in Gram-negatives and teichoic acids (TA) in Gram-positives, both of which play an important factor in surface attachment [8]. For example, TAs were reported to be significant for attachment to plastics and glass found on biomedical devices [16]. Coincidentally, removing TAs in methicillin-resistant *S. aureus* (MRSA) through a *tarO* deletion produced SIPs with reduced attachment strengths across all PEMs compared to the wild type (WT) (Fig 2c).

SIPs continued to be distinguishable at the species level among the clinically significant pathogens (Fig 2a), with differentiating factors including the average magnitude of interaction strengths across PEMs (average SIP), and the variance of interaction strengths across PEMs (SIP spread). Notably, among these strains, *Pseudomonas aeruginosa* displayed the highest average SIP, and vancomycin-intermediate *S. aureus* (VISA) had the smallest SIP spread. Isolating the exact differences in bacterial surface features that produced the observed shifts in SIP is non-trivial and beyond the scope of this initial study. However, experimental clues suggest that differences in SIPs may not be limited to surface chemistry alone. Within the Gram-negative pathogens, *Pseudomonas aeruginosa* displayed significantly higher interaction strengths across all PEMs (Fig 1i) and is unique in its expression of flagella. These hairlike appendages confer swimming motility and increase the probability of surface encounters in *P. aeruginosa* [25,26], likely contributing to its increased average SIP.

Examining other surface features and the genes that control them may be as simple as tracking changes in SIP between a set of mutants and their WT. To investigate further, we compared SIPs of single nucleotide polymorphisms (SNPs) in *S. aureus* with single-point mutations in the *wal* operon, a key regulator of the autolysin-encoding genes in cell wall metabolism [27]. The first case study was *S. aureus* with a single-point mutation in *walR*, which reduced peptidoglycan recycling and increased the cell wall thickness [20]. This was followed by a subsequent single-point mutation in *walK* that restored cell wall metabolism and thickness back to approximately WT levels [20]. The *walR* SIP (Fig 2d) displayed a notable decrease in interaction strength for PEI and PSS and a comparable increase for PDDA, amounting to an overall drop in average SIP compared with the WT. The *walK* SIP reverted to an approximate WT SIP while preserving the order of PDDA and PEI preference from *walR.* In our second case study, cell wall thickness was reverted instead by adding the *mupGmurQmurP* operon encoded on a plasmid (Fig 2e), in effect corroborating the restoration of cell wall metabolism by producing a SIP similar to that of *walK*.

Mutations in the *walKR* system have been linked to multidrug-resistant pathways in *S. aureus* [28]. Reduced susceptibility to vancomycin, a last-line antibiotic used to treat serious MRSA infections [29], is primarily associated with modifications to the cell wall peptide sequence that reduce the binding affinity and action of vancomycin [30]. However, vancomycin-intermediate *S. aureus* (VISA) isolates have also displayed thicker cell walls analogous to the *walR* mutant [12]. Coincidentally, the shifts in SIP from MRSA to VISA (Fig 1m) showed a decrease in average SIP and SIP spread, with drops in PEI and PSS interaction strengths similar to those observed in *walR* (Fig 1k). The more subtle shift in SIP from WT to MRSA (Fig 1m) was not unexpected as the action of methicillin resistance is mainly internal to the cell [31], though minor shifts in wall TA glycosylation have been observed [13]. Very subtle modifications like these could be detectable by improving SIP specificity through an increase in the number and diversity of test surfaces used to provide additional interactions. Regardless, both MRSA and VISA present serious risk factors in clinical settings [32,33] and being able to quickly distinguish between them (and possibly other resistant mutants) in tandem with bacterial identification presents an unignorable clinical potential for SIPs.

**Fig 3:**
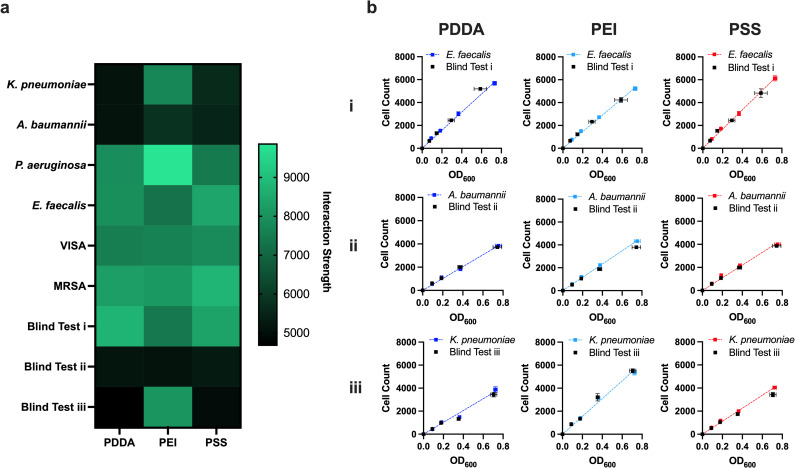
Blind experiment SIP matching. a, All clinically significant pathogen and blind test pathogen SIPs are plotted as a heat map. b, The bacterial-surface attachment curves (surface-adhered cell counts plotted against suspension optical densities) of blind pathogens i, ii, and iii compared with their correctly identified counterparts *E. faecalis*, *A. baumannii*, and *K. pneumoniae*, respectively (mean ± SEM).

In principle, we could determine the identity of an unknown pathogen by matching its SIP to one in a library of pre-measured SIPs. This library would be generated and continuously updated by measuring and compiling the SIPs of all current and new clinically relevant bacterial pathogens and antibiotic-resistant mutants using an optimized protocol developed for a future SIP-based diagnostic. To explore further, three test pathogens were randomly selected, their suspensions prepared blind, and their SIPs measured. We were able to successfully identify each blind test pathogen by comparing and matching SIPs (Fig 3a): i) *Enterococcus faecalis*, ii) *Acinetobacter baumannii*, and iii) *Klebsiella pneumoniae*. The attachment curves and interaction strength values between the blind test pathogens and their correctly identified clinically relevant strain counterparts displayed significant agreement across PEMs (Fig 3b) and were the highest percent matching pairs among all possible SIPs (S2 Table).

## Conclusion

Herein, we introduced a novel technique capable of rapidly differentiating a set of bacteria through their surface interactions, utilizing a quick 10-minute interaction period and only three probing surfaces. By comparing our first-generation SIPs, we were able to successfully distinguish between clinically significant pathogens from Gram stain to individual species. Furthermore, we confirmed the restoration of cell wall metabolism via tracking and comparing SIPs between a series of *S. aureus* mutants in the *walKR* system, broadening the scope and utility of this technique. Lastly, an early glance at its predictive capabilities combined with distinguishing single- and multidrug-resistant forms of *S. aureus* underscored its clinical relevance and future diagnostic potential in combining pathogen identification and AST under a single, rapid, low-cost test.

## Supporting information

S1 Fig All surface-bound bacterial counts per PEM. All bacterial counts plotted against suspension optical densitites (OD_600_) on PDDA (dark blue), PEI (light blue), and PSS (red) PEM surfaces per cell type (N = 15 test bacteria, N = 3 biological replicates per bacteria, N = 120 images counted per bacteria per PEM): a-c, Gram-negative (-) pathogens, d-f, Gram-positive (+) pathogens, g-k, *S. aureus* SNPs, l MRSA SNP, and m-o, blind test pathogens (i: *E. faecalis*, ii: *A. baumanii*, and iii: *K. pneumoniae*).
(TIF)

S2 Fig Attachment curves of all test bacteria. All plots of cell counts per PDDA- (dark blue), PEI- (light blue), and PSS-topped (red) PEM surfaces per cell type (N = 15 test bacteria, N = 540 PEMs) (mean ± SEM): a-c, Gram-negative (-) pathogens, d-f, Gram-positive (+) pathogens, g-k, *S. aureus* SNPs, l MRSA ∆*tarO*, and m-o, blind test pathogens (i: *E. faecalis*, ii: *A. baumannii*, and iii: *K. pneumoniae*).
(TIF)

S3 Fig PEM template and fluid chip assembly. a, A Petri dish lid template outlining the relative positions and dimensions of marked circular boundaries utilized to build each PEM. b, The location, dimensions, and order of double-sided tape addition. c, A schematic displaying the location of added lane toppers (glass cover slips) and lane intlet hydrophobic markings.
(TIF)

S4 Fig PEM assembly rinsing protocol for unidirectional flow. a, The Petri lid was kept at a ~ 35˚ angle during rinsing. b, Surfaces were first lightly rinsed in sequence form left to right (1–3). c, The chip was rotated by 180˚ while maintaining the aforementioned pitch angle. d, The next three surfaces were lightly rinsed in sequence from left to right (4–6). e, The disposal of collected aqueous waste. f, The Petri lid was inverted and tapped (8x) on a lab bench before adding the next polyelectrolyte solution.
(TIF)

S5 Fig Introducing bacterial suspensions to the fluid lanes. An illustration of the protocol used to introduce each concentration of bacterial suspension to an individual fluid lane. a, Fluid lanes containing PEM surfaces were pre-wet with standard concentration PBS buffer. b, The bacterial suspension was introduced to the fluid lane via wicking. c, The bacterial suspension was left to interact with the PEM surface for 10 minutes. d, The fluid lane was rinsed with PBS buffer. e, The surface was imaged using an inverted microscope.
(TIF)

S6 Fig Optical microscopy images. Four images (200x magnification) of PDDA-bound *Staphylococcus aureus* ATCC29213 wild-type bacteria from lowest to highest added suspension concentrations (a-d).
(TIF)

S1 Table All bacteria tested and their suspension optical densities. A list of all bacterial species tested, including their strains and SNPs, Gram stain, and liquid suspension optical densitites (OD_600_) pre- and post-PBS resuspension. Acronyms CA, MRSA, VISA stand for community-associated, methicillin-resistant *Staphylococcus aureus* and vancomycin-intermediate *Staphylococcus aureus*, respectively.
(TIF)

S2 Table SIP percent matching between pathogenic and blind test strains. The percent agreement between pathogenic and blind test strain attachment curve slopes per PEM type, with the correctly identified pathogen highlighted (green): a blind test i, b blind test ii, and c blind test iii.
(TIF)

S1 Spreadsheet All surface-bound cell count data. All tabulated bacterial count data of all PEM-bound test bacteria used to produce all attachment curves and SIPs data.
(XLSX)
