# Supplementary figures and images for: Differentiating bacteria by their unique surface interactions
Source: PLoS One. 2025 Jun 30;20(6):e0327489. doi: 10.1371/journal.pone.0327489 (PMC12208452; doi:10.1371/journal.pone.0327489)

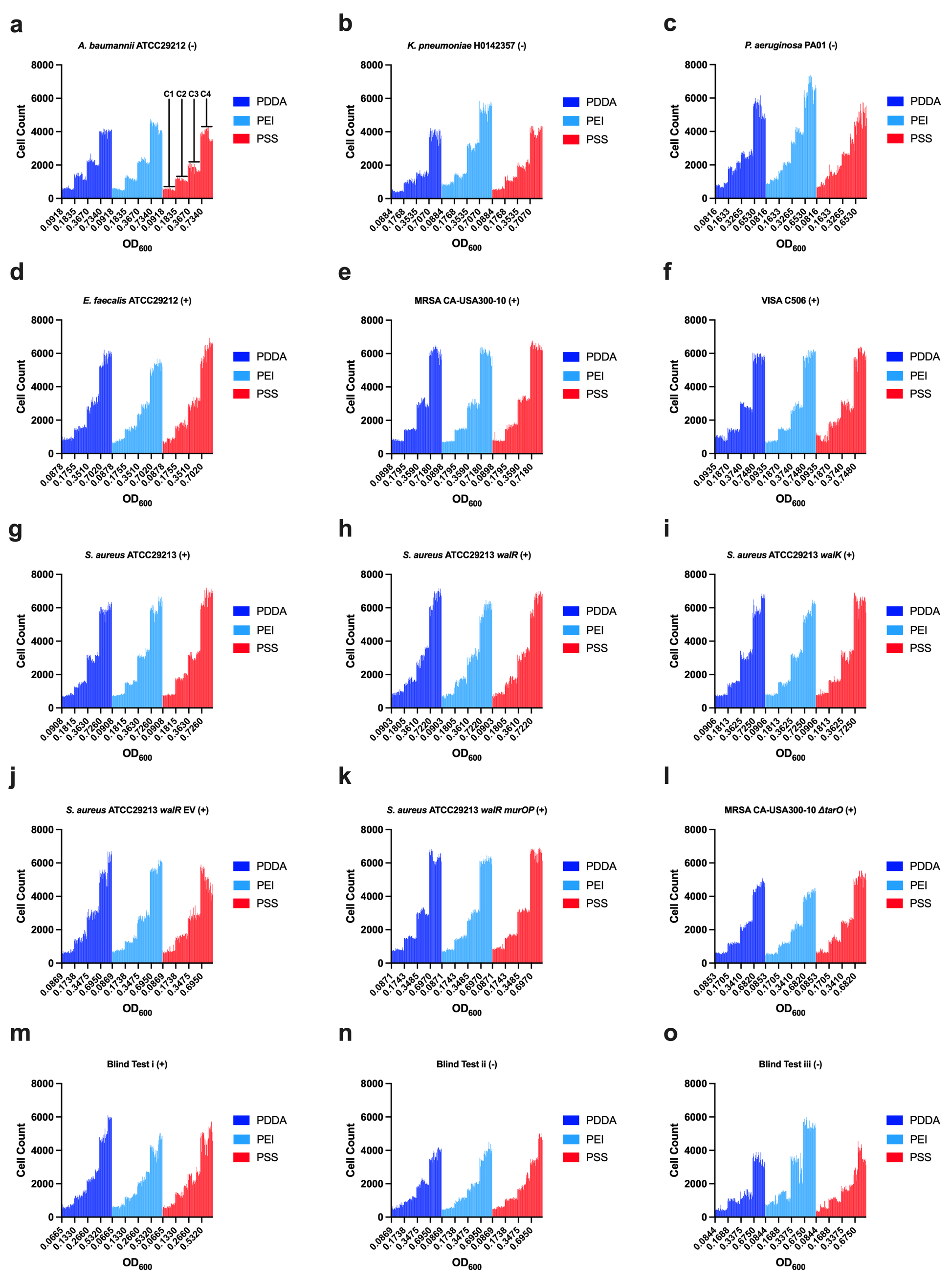

Supplement: S1 Fig — All bacterial counts plotted against suspension optical densitites (OD600) on PDDA (dark blue), PEI (light blue), and PSS (red) PEM surfaces per cell type (N = 15 test bacteria, N = 3 biological replicates per bacteria, N = 120 images counted per bacteria per PEM): a-c, Gram-negative (-) pathogens, d-f, Gram-positive (+) pathogens, g-k, S. aureus SNPs, l MRSA SNP, and m-o, blind test pathogens (i: E. faecalis, ii: A. baumanii, and iii: K. pneumoniae). (TIF) [file pone.0327489.s001.tif]

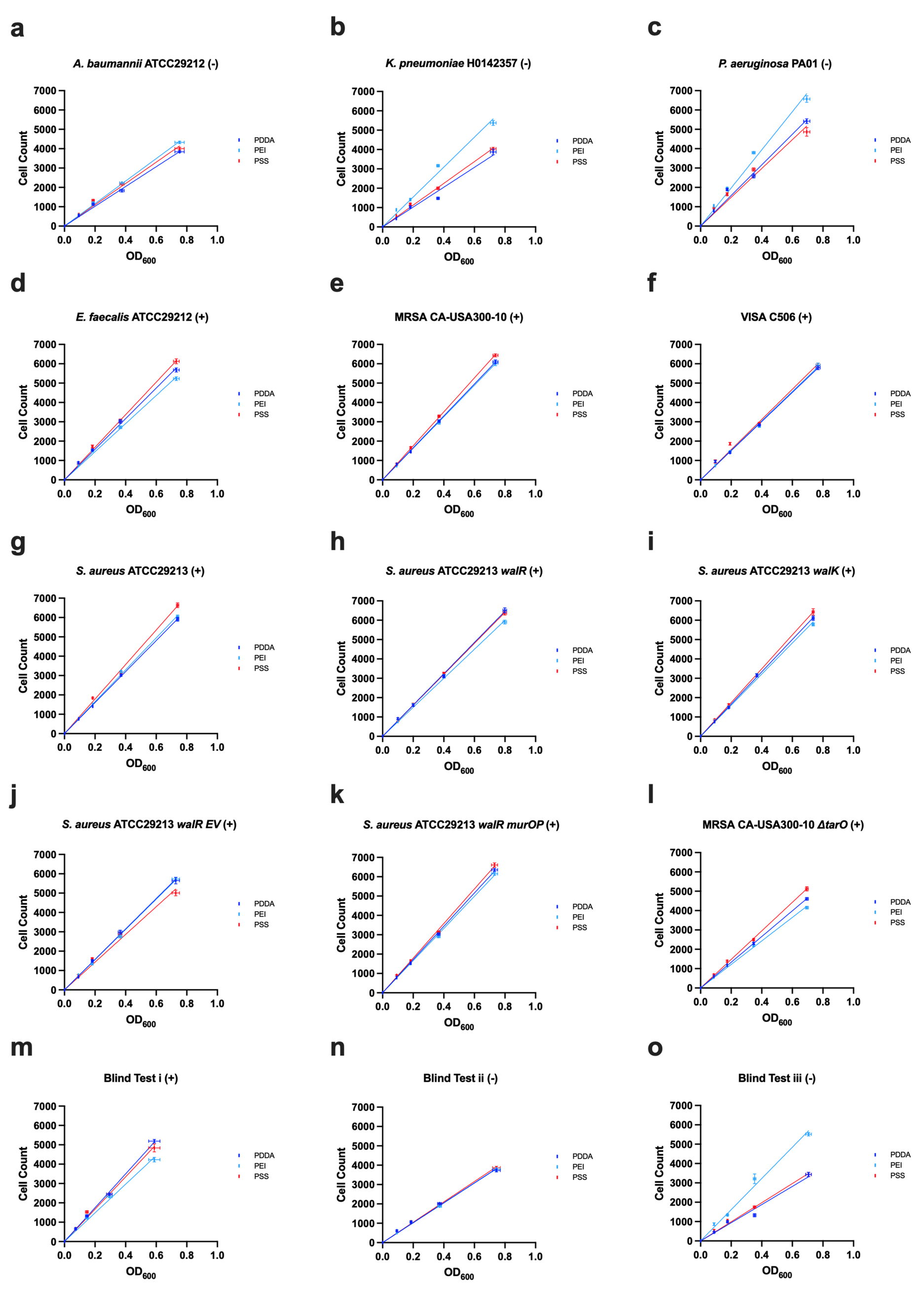

Supplement: S2 Fig — All plots of cell counts per PDDA- (dark blue), PEI- (light blue), and PSS-topped (red) PEM surfaces per cell type (N = 15 test bacteria, N = 540 PEMs) (mean ± SEM): a-c, Gram-negative (-) pathogens, d-f, Gram-positive (+) pathogens, g-k, S. aureus SNPs, l MRSA ∆tarO, and m-o, blind test pathogens (i: E. faecalis, ii: A. baumannii, and iii: K. pneumoniae). (TIF) [file pone.0327489.s002.tif]

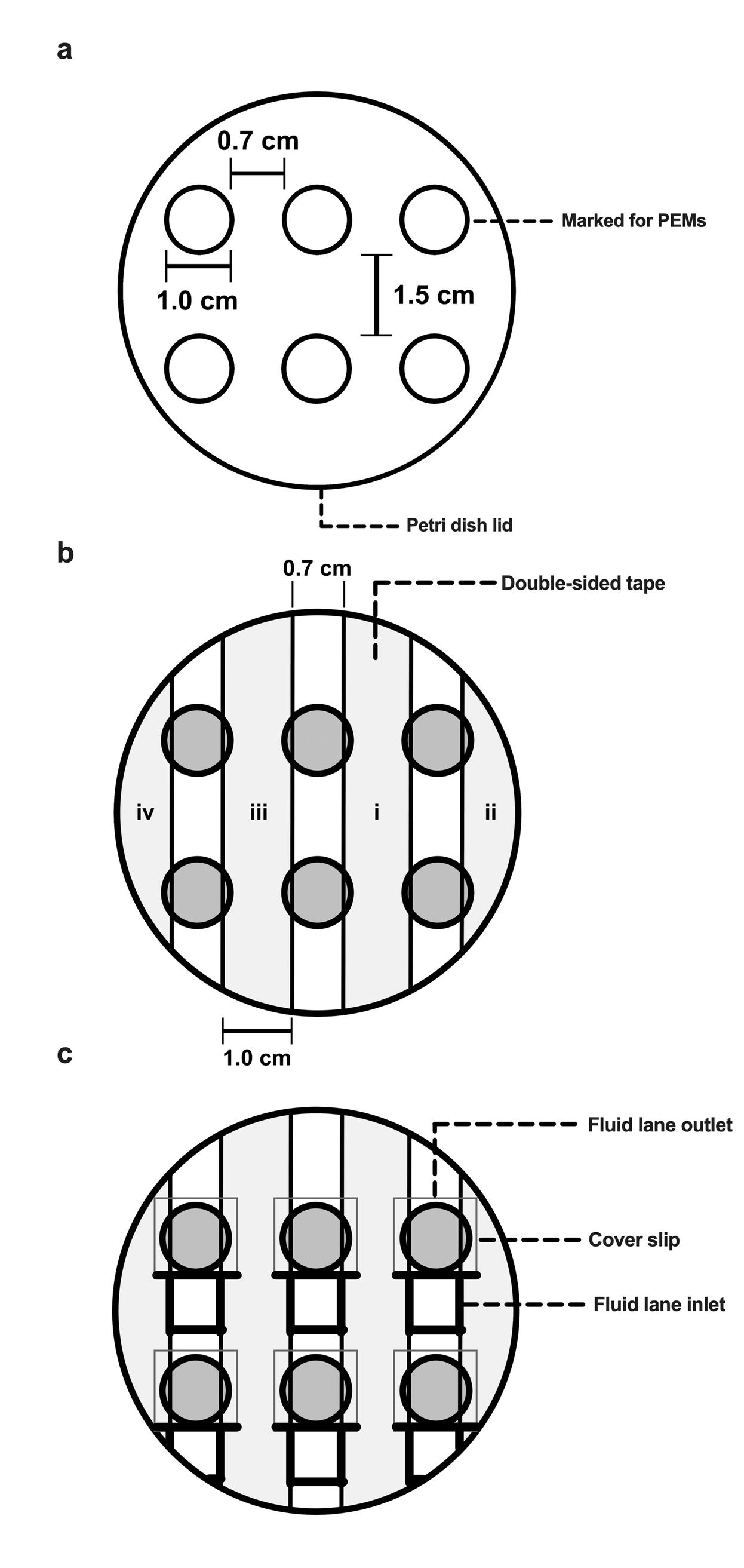

Supplement: S3 Fig — a, A Petri dish lid template outlining the relative positions and dimensions of marked circular boundaries utilized to build each PEM. b, The location, dimensions, and order of double-sided tape addition. c, A schematic displaying the location of added lane toppers (glass cover slips) and lane intlet hydrophobic markings. (TIF) [file pone.0327489.s003.tif]

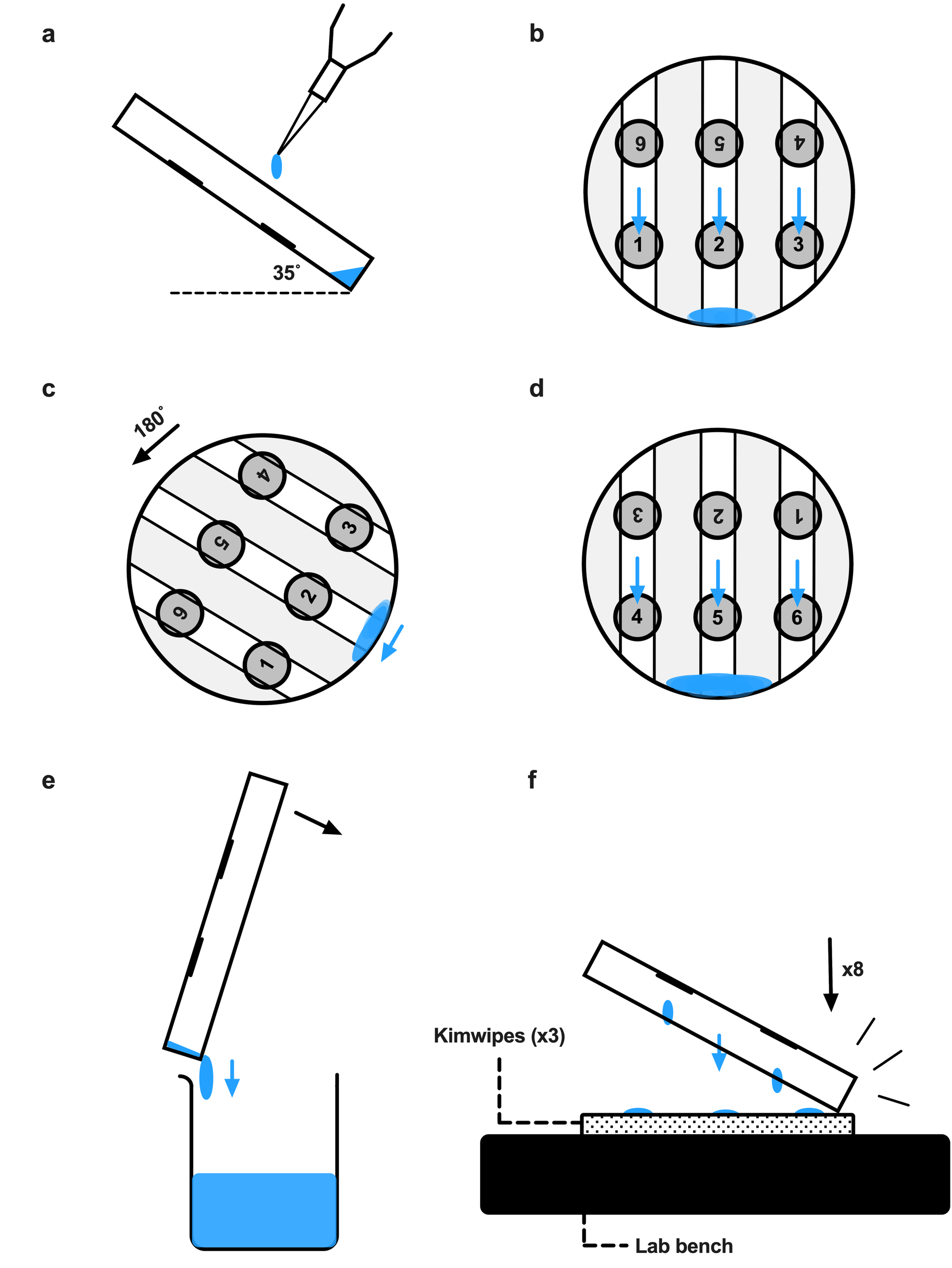

Supplement: S4 Fig — a, The Petri lid was kept at a ~ 35˚ angle during rinsing. b, Surfaces were first lightly rinsed in sequence form left to right (1–3). c, The chip was rotated by 180˚ while maintaining the aforementioned pitch angle. d, The next three surfaces were lightly rinsed in sequence from left to right (4–6). e, The disposal of collected aqueous waste. f, The Petri lid was inverted and tapped (8x) on a lab bench before adding the next polyelectrolyte solution. (TIF) [file pone.0327489.s004.tif]

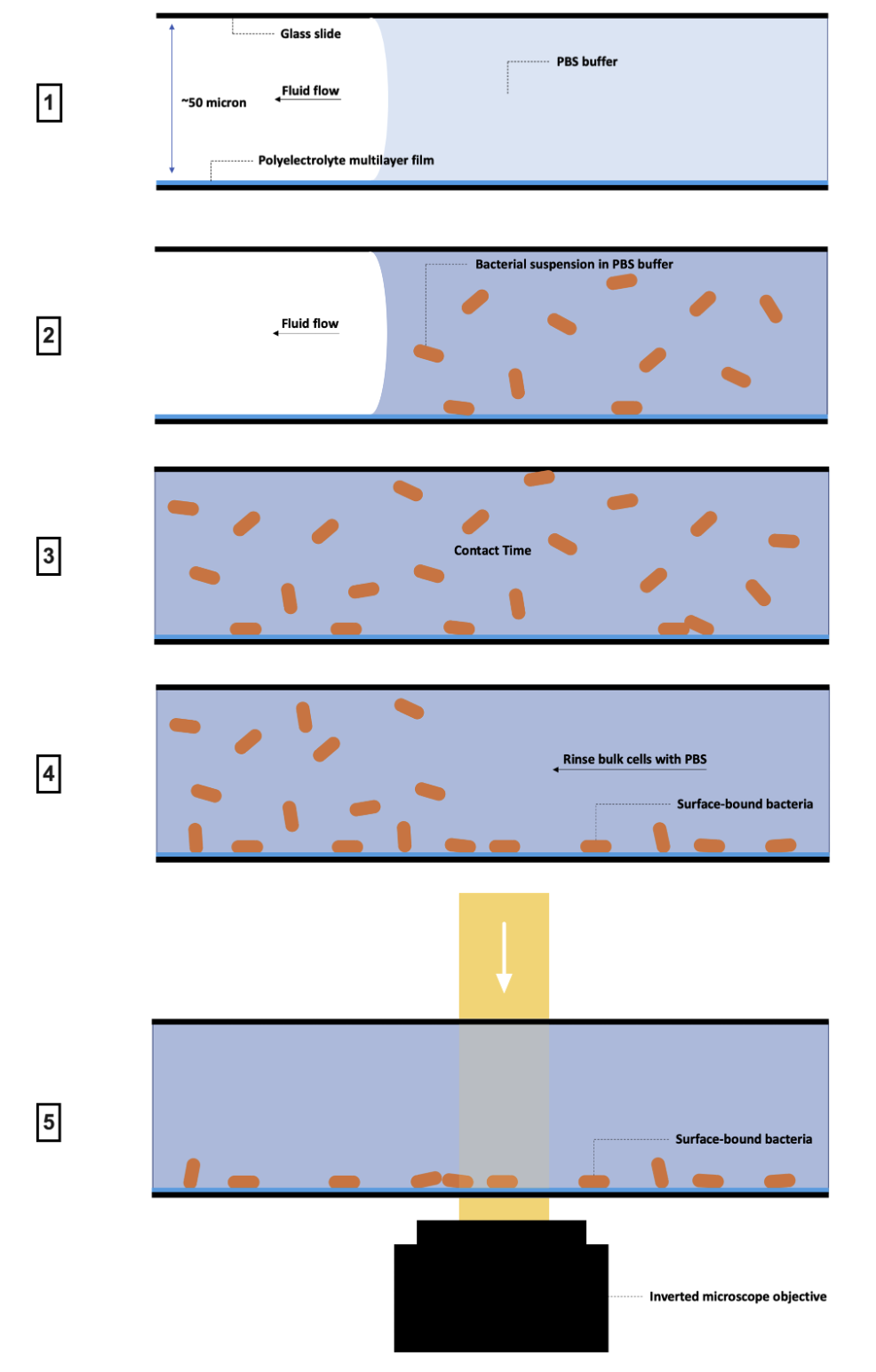

Supplement: S5 Fig — An illustration of the protocol used to introduce each concentration of bacterial suspension to an individual fluid lane. a, Fluid lanes containing PEM surfaces were pre-wet with standard concentration PBS buffer. b, The bacterial suspension was introduced to the fluid lane via wicking. c, The bacterial suspension was left to interact with the PEM surface for 10 minutes. d, The fluid lane was rinsed with PBS buffer. e, The surface was imaged using an inverted microscope. (TIF) [file pone.0327489.s005.tif]

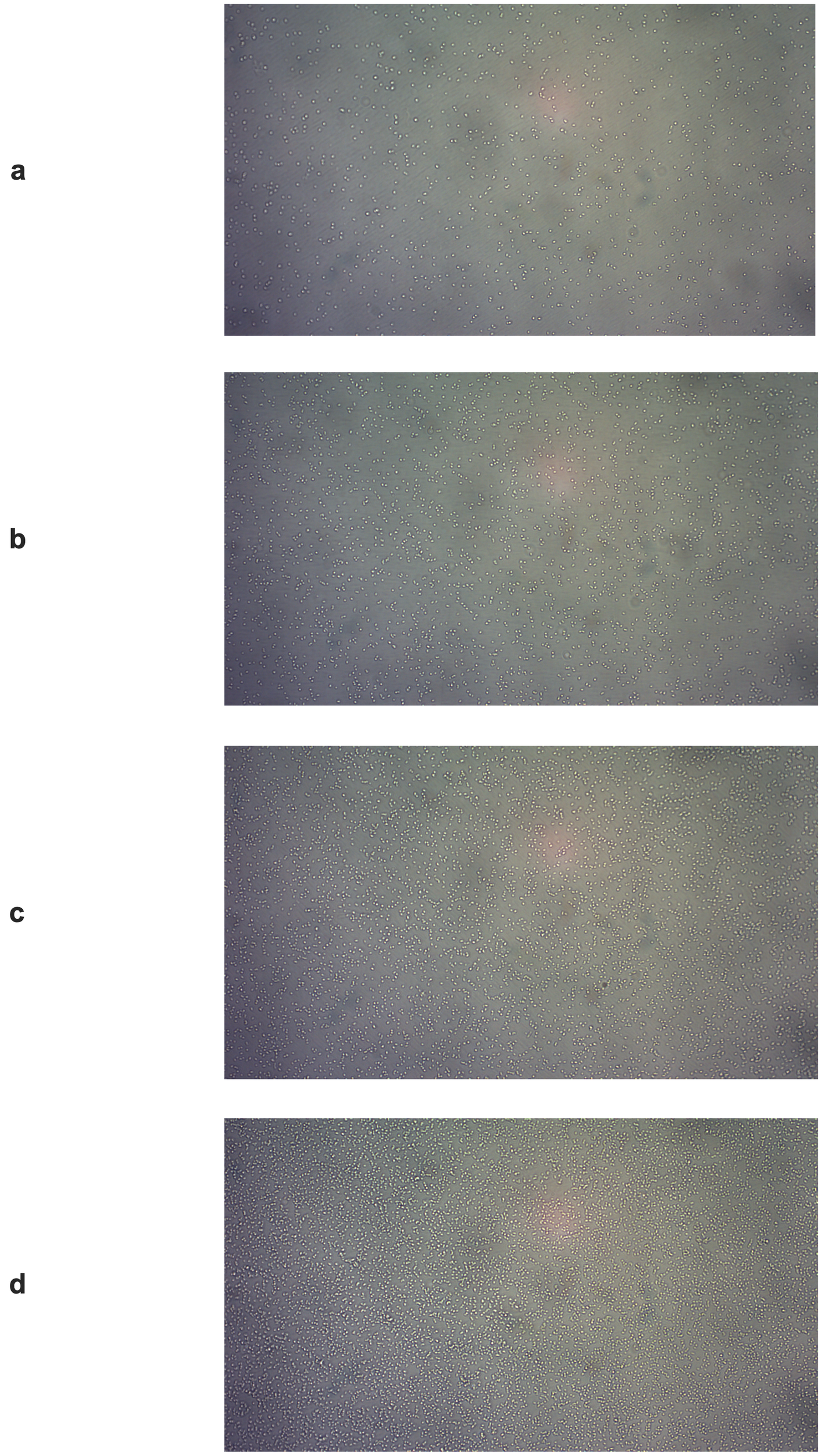

Supplement: S6 Fig — Four images (200x magnification) of PDDA-bound Staphylococcus aureus ATCC29213 wild-type bacteria from lowest to highest added suspension concentrations (a-d). (TIF) [file pone.0327489.s006.tif]

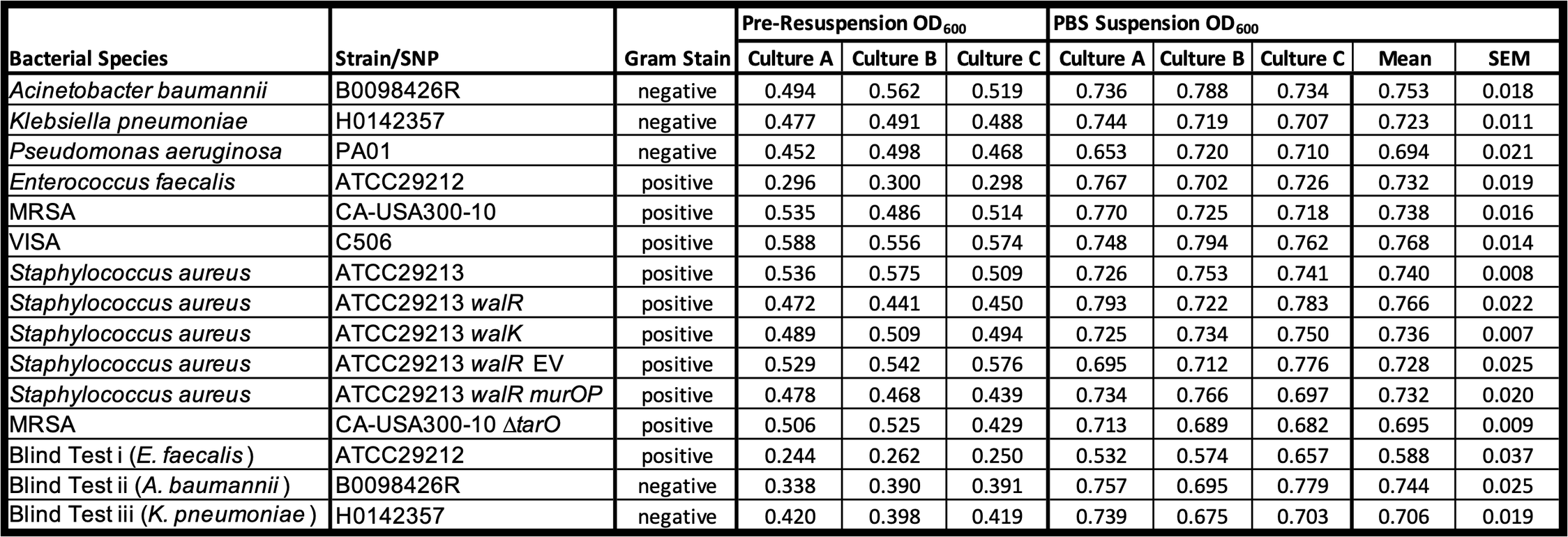

Supplement: S1 Table — A list of all bacterial species tested, including their strains and SNPs, Gram stain, and liquid suspension optical densitites (OD600) pre- and post-PBS resuspension. Acronyms CA, MRSA, VISA stand for community-associated, methicillin-resistant Staphylococcus aureus and vancomycin-intermediate Staphylococcus aureus, respectively. (TIF) [file pone.0327489.s007.tif]

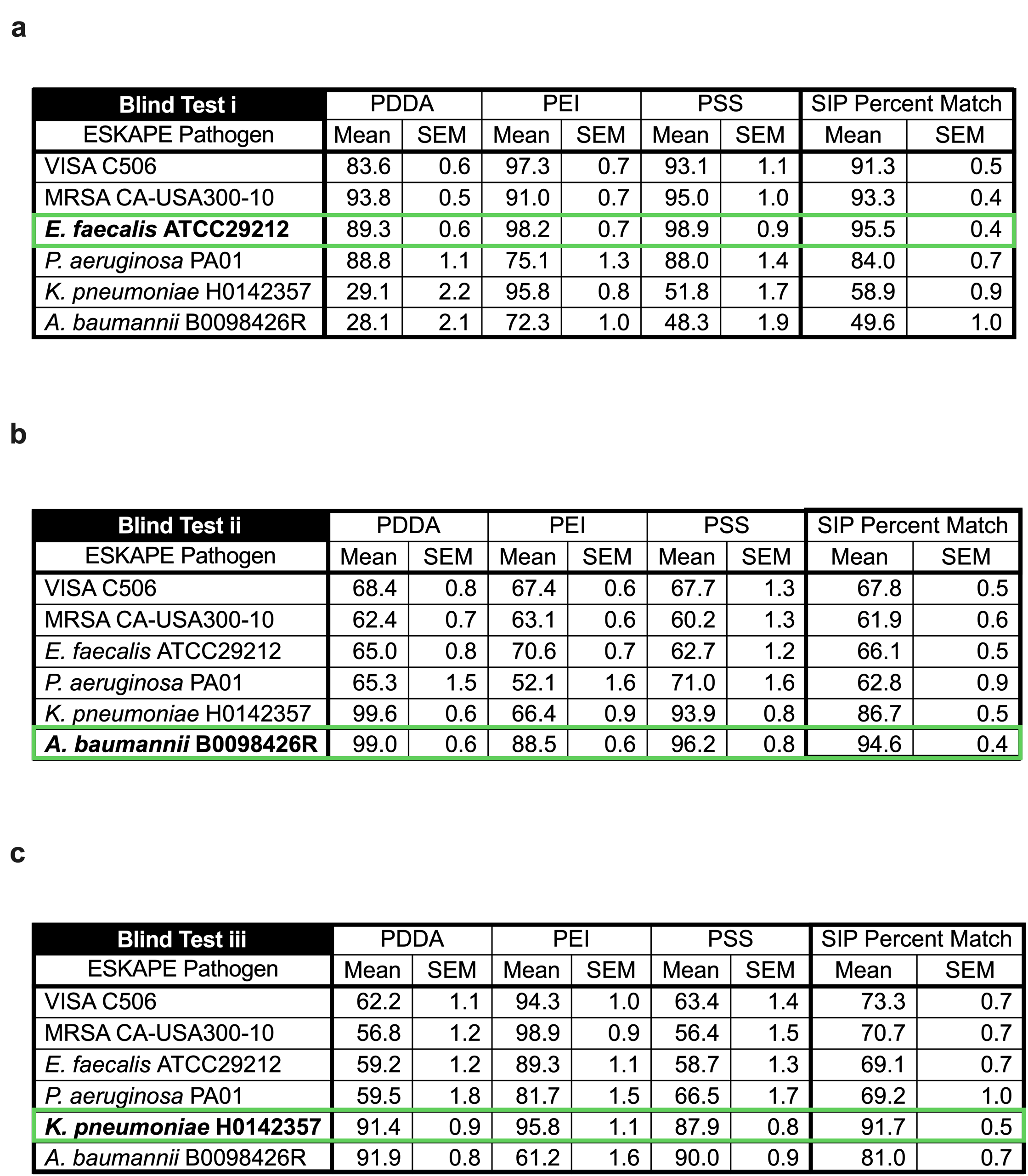

Supplement: S2 Table — The percent agreement between pathogenic and blind test strain attachment curve slopes per PEM type, with the correctly identified pathogen highlighted (green): a blind test i, b blind test ii, and c blind test iii. (TIF) [file pone.0327489.s008.tif]
